# Photocatalytic Hydrogen Production Using Porous 3D Graphene-Based Aerogels Supporting Pt/TiO_2_ Nanoparticles

**DOI:** 10.3390/gels8110719

**Published:** 2022-11-07

**Authors:** Márta Kubovics, Cláudia G. Silva, Ana M. López-Periago, Joaquim L. Faria, Concepción Domingo

**Affiliations:** 1 Instituto de Ciencia de Materiales de Barcelona, CSIC, Campus UAB s/n, 8193 Bellaterra, Spain; 2 LSRE-LCM-Laboratory of Separation and Reaction Engineering–Laboratory of Catalysis and Materials, Faculty of Engineering, University of Porto, Rua Dr. Roberto Frias, 4200-465 Porto, Portugal; 3 ALiCE-Associate Laboratory in Chemical Engineering, Faculty of Engineering, University of Porto, Rua Dr. Roberto Frias, 4200-465 Porto, Portugal

**Keywords:** aerogel, graphene oxide, supercritical CO_2_, photocatalysis, H_2_ production

## Abstract

Composites involving reduced graphene oxide (rGO) aerogels supporting Pt/TiO_2_ nanoparticles were fabricated using a one-pot supercritical CO_2_ gelling and drying method, followed by mild reduction under a N_2_ atmosphere. Electron microscopy images and N_2_ adsorption/desorption isotherms indicate the formation of 3D monolithic aerogels with a meso/macroporous morphology. A comprehensive evaluation of the synthesized photocatalyst was carried out with a focus on the target application: the photocatalytic production of H_2_ from methanol in aqueous media. The reaction conditions (water/methanol ratio, catalyst concentration), together with the aerogel composition (Pt/TiO_2_/rGO ratio) and architecture (size of the aerogel pieces), were the factors that varied in optimizing the process. These experimental parameters influenced the diffusion of the reactants/products inside the aerogel, the permeability of the porous structure, and the light-harvesting properties, all determined in this study towards maximizing H_2_ production. Using methanol as the sacrificial agent, the measured H_2_ production rate for the optimized system (18,800 µmol_H2_h^−1^g_NPs_^−1^) was remarkably higher than the values found in the literature for similar Pt/TiO_2_/rGO catalysts and reaction media (2000–10,000 µmol_H2_h^−1^g_NPs_^−1^).

## 1. Introduction

Hydrogen (H_2_) is one of the most promising carbon-neutral alternatives as a renewable energy source, mainly due to its high calorific value and attainable purity [1]. Large-scale H_2_ production via photocatalytic water splitting is a simple and cheap method, although the low reached conversion values (ca. 1%) cause the procedure to still be inefficient and economically unviable [2]. Consequently, the study of new catalytic systems that enhance the conversion is important to improve the efficiency and sustainability of the process [3]. Heterogeneous photocatalysis, working via water splitting or by the photoreforming of organic waste, is an attractive solution for H_2_ production, since the utilization of solar energy moderates urgent environmental and energy issues [4].

In the photocatalytic H_2_ production process, a light beam with sufficient energy irradiates a semiconductor material. The thus attained excited electrons (e^−^) and holes (h^+^) migrate onto the surface of the catalyst and act as reducing and oxidizing agents, respectively. Hence, the reduction and oxidation potentials of the reactant (e.g., water, alcohol, glycerol) must be within the band gap of the photocatalyst [5]. Semiconductors, such as titanium dioxide (TiO_2_), cadmium sulfide (CdS), and carbon nitride (C_3_N_4_), fulfill this condition and are often used as photocatalytic systems [6,7,8]. TiO_2_ is traditionally one of the most utilized semiconductors owing to its highly negative conduction band potential, and thus strong reduction ability. This oxide is chemically stable, cheap, and abundant. However, its use also experienced some drawbacks. First, due to its wide band gap, UV light is necessary for activation, and this light is present in a percentage lower than 5% in sunlight [9]. Second, TiO_2_ experienced an extremely rapid recombination rate of the photogenerated h^+^ and e^−^, in the order of 10^−12^ − 10^−11^ s, while a value in the interval of 10^−9^ − 10^−7^ s is required for capturing the generated species in a successful redox reaction [7]. To improve the photocatalytic efficiency, the semiconductor must be combined with agents that can scavenge the photogenerated e^−^, e.g., Pt [1,10]. It is worth mentioning that Pt has been recently included in the list of “critical raw materials -with economic importance, but high supply risk-” by the European Commission [11]. The target objective must be to decrease our dependence on these critical raw materials by minimizing their percentage of use in the designed product [12].

A newly designed and feasible strategy to moderate the detrimental effects of the large band gap and high e^−^/h^+^ recombination rate of TiO_2_ is to incorporate graphene into the catalyst [13]. Undoubtedly, 2D graphene sheets are becoming a top choice as catalyst compartment/supports due to their unique physicochemical properties, related to their large surface area, high thermal and electrical conductivity, and ability to tailor the band gap energy level of the semiconductor [14,15,16]. Furthermore, due to its high work function, e^−^ from the conduction band of the semiconductor can be accepted and transferred by the graphene [17]. In parallel, the 2D sheets ensure an appropriate surface for extensively anchoring the semiconductor in the form of nanoparticles (NPs), which can be deposited on both sides of exfoliated graphene flakes, displaying the end composite with a high concentration of active sites for the catalytic reaction [18,19]. The main drawback of using this support is the large tendency of 2D graphene flakes to aggregate, which results in the poor accessibility of the reagents and the light to the catalytically active NPs trapped between the flakes, e^−^ transport hindering, and the poor diffusion of the gaseous product [7,20,21]. The penetration depth of the UV light in TiO_2_ is limited to ca. 100 nm; thus, the formation of large aggregates of NPs would result in an increased amount of semiconductors not affected by the radiation [22]. Several works can be found in the literature using composites of (Pt)TiO_2_ NPs and reduced graphene oxide (rGO) [22,23,24,25,26,27,28]. These systems are composed of either multiple stacked layers of rGO covered with NP agglomerates [26,27] or photoactive NPs covered with an rGO layer [28]. In other works, the aggregation of the Pt/TiO_2_ NPs has been directed to build 3D porous composites with rGO added as an additive [29,30]. 

In this study, a different approach is presented to build a 3D structure, in which an rGO aerogel matrix provides support for the photoactive NPs: Pt/TiO_2_@rGO. The precursor is a 3D aerogel structure of graphene oxide (GO): Pt/TiO_2_@GO. GO is a highly oxygenated precursor, with mainly hydroxyl, epoxy, and carboxylic functionalities, easily exfoliated in polar liquids, and capable of establishing strong metal–support interactions to ensure NP dispersion and to avoid NP leaching during catalytic reaction [31,32,33]. As with other common aerogels [34,35], those of GO have a low-density network with a meso/macroporous structure. The Pt/TiO_2_@GO intermediate composite was synthesized in the form of a monolith using a previously described one-pot supercritical CO_2_ (scCO_2_) methodology [36]. After aerogel synthesis, the number of oxygenated groups on the support can be modulated by thermal treatment to prepare the desired Pt/TiO_2_@rGO end product [37]. The macroscopic size and variable shape of the synthesized 3D aerogel macrostructures bring advantages of operability and recoverability. The obtained aerogel composites were structural and texturally characterized. Moreover, the new catalytic system was evaluated for its photocatalytic H_2_ production in aqueous methanol solutions. The process was adjusted for effective H_2_ production regarding the reaction conditions (catalyst concentration, composition of reaction mixture), catalyst composition (Pt:TiO_2_:rGO ratios), and architecture (one-piece monolith or smashed aerogel). Optimizing the Pt/TiO_2_@rGO composite leads to H_2_ production rates in an aqueous methanol solution of ca. 2–10 times higher than the values reported for similar systems in the literature [25,29]. 

## 2. Results and Discussion

### 2.1. Aerogel Synthesis

**Figure 1:**
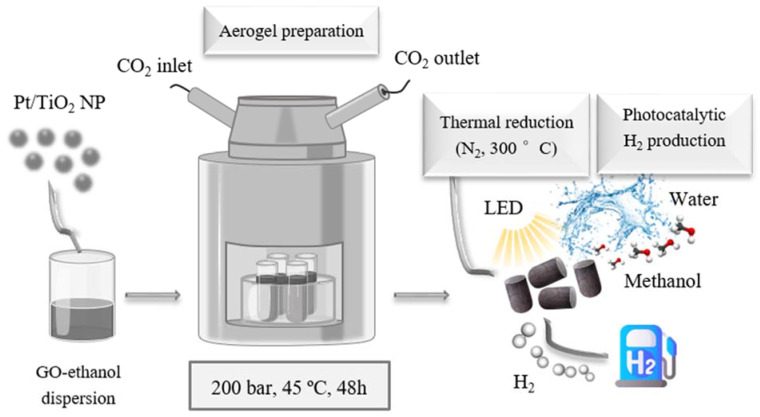
Scheme of the scCO_2_-assisted synthesis for Pt/TiO_2_@rGO aerogels.

The xPt/TiO_2_@rGO composite aerogels were synthesized through the intermediate xPt/TiO_2_@GO aerogel (Figure 1), involving a non-reduced GO matrix containing a high amount of oxygenated functional groups, mainly hydroxyl, epoxy, and carboxylic, which are located on the basal plane and at the edges of the 2D platelets. The oxygenated functionalities facilitate the dispersion and exfoliation of GO in aqueous and polar solutions via simple sonication. Moreover, preserving the oxygenated functional groups in GO during scCO_2_ aerogel synthesis was essential to ensure the presence of many anchoring points on the substrate for the NPs, which guarantees the establishment of strong interactions with a net or composed hydrophilic TiO_2_ involving hydroxyl groups on the surface [32,38,39]. Three different compositions for the NPs in the intermediate were tested, namely, 1Pt/TiO_2_@GO, 0.5Pt/TiO_2_@GO, and 0.1Pt/TiO_2_@GO, corresponding to an initially mixed amount of Pt with the TiO_2_ NPs of 1, 0.5 and 0.1 wt%, respectively. To activate the aerogel for the catalytic process, the xPt/TiO_2_@GO intermediate samples were exposed to a temperature of 300 °C in a N_2_ atmosphere. This treatment eliminates most of the oxygenated groups in GO. The reduction step is crucial to further achieve an efficient photocatalytic reaction, since important graphene-like characteristics, such as high e^−^ mobility, are partially restored by removing some of the oxygenated groups. Hence, the rGO matrix can act as an efficient sink, where the photogenerated e^−^ are stored and transferred [13,40]. During reduction, ca. 30 wt% is eliminated from the sample, corresponding mostly to oxygenated functionalities. Taking this into account, the estimated ratios of the NPs:rGO phase in the xPt/TiO_2_@rGO samples were calculated as 3:1 and 9:1, corresponding to the intermediates with NPs:GO ratios of 2:1 and 6:1, respectively. The Pt content in the reduced composites was measured by ICP-MS, giving values close to the expected quantity, 0.9, 0.5, and 0.1 wt%, percentages calculated concerning TiO_2_ weight. Thus, practically, no noble metal loss occurs during the preparation procedure. The obtained samples were named as 0.9Pt/TiO_2_@rGO, 0.5Pt/TiO_2_@rGO, and 0.1Pt/TiO_2_@rGO.

### 2.2. Aerogels Structure

**Figure 2:**
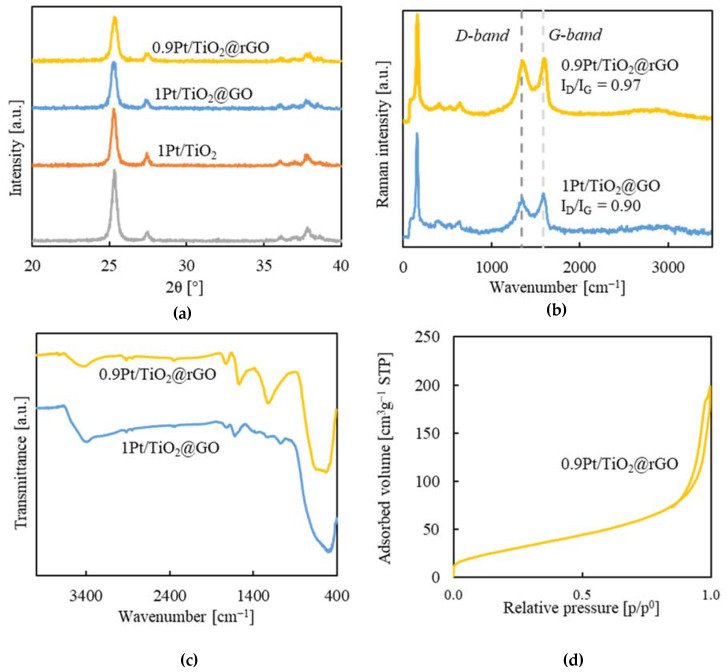
Structural characterization of bare NPs and as-synthesized or reduced aerogel composite for the 1Pt/TiO_2_@GO (2:1) and 0.9Pt/TiO_2_@rGO (3:1) samples: (**a**) PXRD patterns, (**b**) Raman spectra, in which D and G bands are indicated with dashed lines, (**c**) FTIR spectra, and (**d**) N_2_ adsorption/desorption analysis of the reduced aerogel.

The composite components, as well as the intermediate xPt/TiO_2_@GO and reduced xPt/TiO_2_@rGO aerogels, were structurally analyzed by PXRD. Figure 2a shows the main signals in the patterns obtained in the 2θ interval of 20 to 40°, with the lines corresponding to anatase (2θ = 25.4, 37.0, 37.9, and 38.7°) and rutile (2θ = 27.4 and 36.2°), which were identical in the bare TiO_2_ P25 and composed Pt/TiO_2_ patterns. The signal of GO is described to appear at low angles, ca. 11° [41]. This signal could be observed for the intermediate non-reduced sample as a minor peak at this 2θ (Figure S1), while it disappears from the pattern of the reduced composite. The broadening of the diffraction lines was used to estimate NPs diameter by using the Scherrer equation. For bare TiO_2_ and binary xPt/TiO_2_ NPs, a size of ca. 21–22 nm was estimated, similar to that observed with TEM microscopy (Figure S2a,b). The NPs size in the reduced composites was similar, ca. 19–20 nm. The estimated particle size was, in all cases, in the range of the mean value given for the commercial TiO_2_ P25 (ca. 20 nm). Consequently, no significant alteration of the crystalline structure or in the particle size of the TiO_2_ took place throughout the deposition of Pt on its surface or the during aerogel formation and reduction. 

Raman spectroscopy was used to investigate the structural changes occurring in the GO functional groups during the reduction process of the aerogel. The as-synthesized and reduced aerogels recorded spectra were characterized by the presence of the typical G and D bands at 1590 and 1345 cm^−1^, respectively (Figure 2b). The G band is generated by the in-plane vibrations of sp^2^-bonded carbon atoms (C–C stretching), while the D-band represents the out-of-plane sp^3^ vibrations corresponding to the defects in the graphitic structure. The ratios of the intensities of the D and G bands (I_D_/I_G_) were 0.90 and 0.97 for the GO and rGO composite aerogels, respectively. This result indicates that both synthesized and reduced samples have an elevated degree of sp^3^ defects in the graphitic structure. For the xPt/TiO_2_@GO samples, the defects represent the highly oxygenated character of GO. For the xPt/TiO_2_@rGO composites, defects not only originated from the residual oxygenated groups but there are also structural defects (holes, vacancies, dislocations, etc.) caused by the thermal treatment applied for sample reduction. It has been described that reduction under relatively mild conditions, such as the ones used in this work, triggers the formation of these defects in rGO [42]. A considerable number of new sp^2^ graphitic domains are formed, but of small size [43]. Moreover, a broadened band observed in the 2700–3000 cm^−1^ region of the Raman spectra, the usual position of the 2D peak in graphene, is indicative of randomly oriented multilayer graphene composing the aerogels [44]. Finally, the presence of the NPs in the composite was noticed by the detection of the TiO_2_ bands at 151, 394, 515, and 630 cm^−1^. The oxygenated character of GO in the intermediate composite and reduced aerogel was further analyzed by FTIR spectroscopy. The spectra of the intermediate samples (Figure 2c) show the GO functional groups by displaying the vibrational modes of C=O at 1719 cm^−1^, C-OH at 1221 cm^−1^, C-O at 1060 cm^−1^, C-O-C at 1370 cm^−1^, and OH at 1618 and 3400 cm^−1^, the latter indicating also adsorbed water. The reduced sample displayed an intense C=C band at 1550 cm^−1^, indicating the partial restoration of the graphitic structure. However, the bands of most of the oxygen groups in GO were somehow preserved in the rGO samples, only the peaks corresponding to epoxy vanished totally, while the peak at 3400 cm^−1^ corresponding to the hydroxyl groups diminished. The presence of the NPs in the composites is shown by the intense and broad stretching band appearing at 500–800 cm^−1^. Raman and FTIR characterization indicate that, under the used experimental conditions for the reduction, 300 °C and a N_2_ atmosphere, the GO phase was partially reduced, but a significant amount of oxygenated functionalities was conserved in the structure of rGO.

### 2.3. Textural Properties and Morphology

The textural properties of the synthesized aerogels were analyzed by N_2_ adsorption/desorption at low temperatures. Figure 2d shows the isotherm recorded for the sample 0.9Pt/TiO_2_@rGO (3:1), representative of all the studied systems, while the isotherms of the precursors can be found in the SI (Figure S3). The isotherm of the 0.9Pt/TiO_2_@rGO is described as type IV at low and medium relative pressure and type II at high relative pressure, which is characteristic of nanoporous structures with both meso- and macropores and a negligible contribution of microporosity. A similar shape was found for the non-reduced GO precursor, while the 0.9Pt/TiO_2_ NPs constitute a mesoporous system originated by particle aggregation. The S_a_ value for the 0.9Pt/TiO_2_@rGO sample was in the order of 110 m^2^g^−1^. This value is inferior to that found in the non-reduced GO sample (*ca.* 150 m^2^g^−1^) but is superior to pristine NPs (ca. 50 m^2^g^−1^). Drying gels with scCO_2_ is known to produce relatively denser aerogels than drying at the critical point of the alcohol due to moderate shrinkage occurring upon gelation and drying. In this work, a diameter of ca. 0.8 cm was measured for the cylindrical aerogel intermediates xPt/TiO_2_@GO, indicating that they suffered some contraction in the axial direction since they were synthesized in a vial of 1 cm diameter. Further, some extra tightening occurs during reduction, leading to monoliths of ca. 0.7 cm diameter for the end products xPt/TiO_2_@rGO. Aerogels with mesoporosity homogeneously distributed along all the mesopore range were obtained (Figure S4), with a BJH V_p_ of 0.30 cm^3^g^−1^ and an average mesopore size of 10 nm.

**Figure 3:**
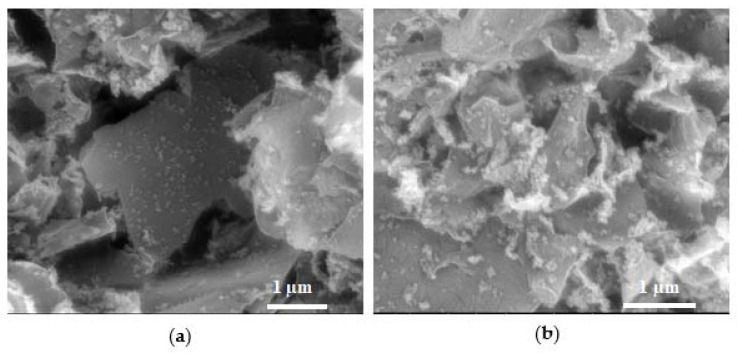
SEM images of samples: (**a**) 1Pt/TiO_2_@GO (2:1), and (**b**) 0.9Pt/TiO_2_@rGO (3:1), representative of the morphology of as-synthesized and reduced aerogels.

All the different xPt/TiO_2_@GO synthesized intermediate aerogels have similar morphology; they are highly porous with a sponge-like macrostructure, as shown in the SEM image of Figure 3a. The SEM images of the reduced monoliths displayed, similarly to the intermediates, a 3D structure with interconnected meso- and macropores (Figure 3b). 

**Figure 4:**
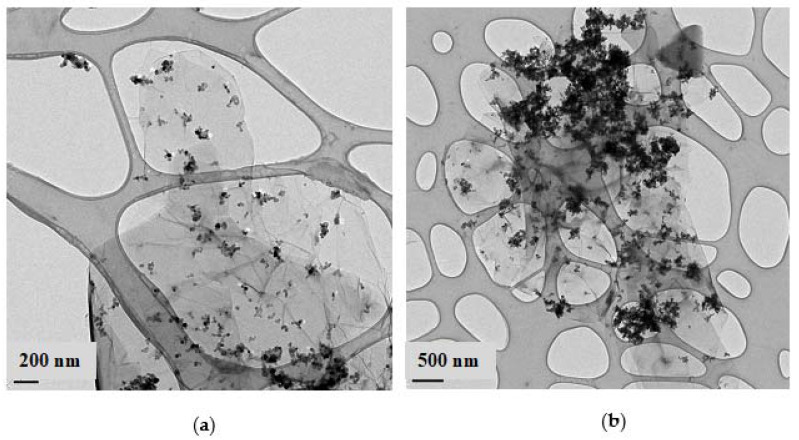
TEM images of the 0.9Pt/TiO_2_@rGO samples with NPs:rGO ratios: (**a**) 3:1 and (**b**) 9:1.

In most of the as-synthesized and reduced samples, the NPs can be discerned as a highly dispersed phase deposited on the surface of the GO or rGO plates (Figure 4a). However, the formation of large aggregates was also detected for the samples with the highest NPs:rGO (9:1) ratio (Figure 4b). 

### 2.4. Aerogels Optical Properties

**Figure 5:**
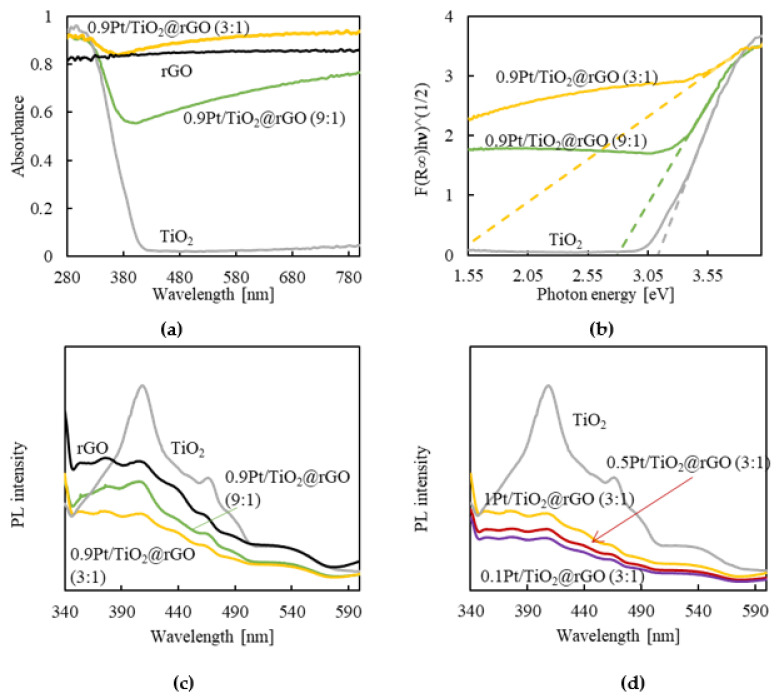
Optical characterization of bare NPs and reduced aerogel composites: (**a**) UV-VIS diffuse reflectance spectra, (**b**) band gap energy determined from the Tauc plot (the linear part of the plot is extrapolated to the *x*-axis), (**c**) photoluminescence spectra at an excitation wavelength of 320 nm, and (**d**) samples of xPt/TiO_2_@rGO (3:1) with different Pt ratios.

The optical properties of the reduced composites, as well as those of bare TiO_2_ and rGO, were analyzed by UV-VIS diffuse reflectance spectroscopy to investigate the samples’ photoresponse (Figure 5a). TiO_2_ was active in the UV zone and exhibited an abrupt absorption edge around 400 nm, while rGO displayed a continuous absorption in the visible range. For the reduced composites, a broad background absorption in the visible range was observed as a consequence of rGO black characteristics, more notable in the sample with the lowest percentage of NPs, e.g., xPt/TiO_2_@rGO (3:1). The contribution of the NPs to the absorption in the UV zone can be clearly appreciated in the composites’ spectra, although with a red shift in the adsorption edge that was slight, to 450 nm, for the (9:1) composite and pronounced, to 750 nm, when the amount of rGO was increased in the (3:1) sample. This shift indicates an increased photoresponse in the visible range of the composite aerogels with respect to net TiO_2_ NPs. To study the indirect optical band gap of the photocatalyst, a Tauc plot was determined, calculated from the UV-VIS absorption spectrum (see the detailed description in the SI) (Figure 5b) [45,46]. For the bare TiO_2_, a band gap energy of 3.1 eV was estimated from the *x*-axis intercept of the extrapolated line fitted to the linear region of the plot. The effect of the Pt content was analyzed for the xPt/TiO_2_@rGO (3:1) composites with an x range of 0.9–0.1 wt%. The three measured bandgap values (at Pt contents of 0.9, 0.5, and 0.1 wt%) were of ca. 1.5 eV, thus demonstrating the low influence of this parameter. Contrarily, enhancing the ratio of NPs:rGO in the 0.9Pt/TiO_2_@rGO sample from 3:1 to 9:1 results in an increase in the bandgap from 1.5 to 2.8 eV, with a concomitant increase in the transition energy of the photoexcited electrons. This phenomenon is assigned to the generation of impurity energy levels above the valance band in the NPs upon their incorporation onto the rGO surface. Thus, for the excitation of the charge carriers, less energy is required [47]. It is worth mentioning that the shift in the absorption edge and the decrease in the band gap energy are both more notable in the studied compounds than in similar published systems, reporting shift values of only 0.1–0.4 eV [17,26]. This important result is explained by the formation of a large number of Ti–O–C bonds in the xPt/TiO_2_@rGO samples, established between the surface of the TiO_2_ and the rGO flakes [48], and occurring during the reduction and elimination of water from the pre-settle hydrogen bond interactions Ti–OH...OH–GO in the xPt/TiO_2_@GO intermediates. Although the low band gap energy in the (3:1) aerogel suggests a high photoresponse, this feature does not necessarily mark out the best photocatalyst system, since other factors should be taken into account. Importantly, using a high amount of rGO can evoke some activity loss, as the dark flakes can shield some active NPs, such that not all catalytic units are exposed to the light. Hence, to design the right catalyst, a compromise must be attained between the percentage of components (NPs:rGO) in the composite; on the one hand, to increase the band gap through the decrease in the number of NPs and, on the other hand, to reduce darkness via a decrease in the proportion of rGO. 

Photoluminescence experiments were carried out to study the recombination rate of the photogenerated e^−^/h^+^ pairs in the aerogel catalyst. One of the main drawbacks described for the use of TiO_2_ semiconductors in photocatalytic processes is the fast recombination of the photogenerated species [7]. This behavior is clearly evidenced in the photoluminescence spectrum with an intense emission after the photoexcitation of the bare NPs under UV light at 320 nm (Figure 5c). A wide luminescence band is observed for TiO_2_, with a maximum at 410 nm (close to the band gap energy of TiO_2_), which is followed by a less intense signal at 468 nm. The spectra of the reduced xPt/TiO_2_@rGO (3:1) and (9:1) composites did not have the same pattern as that of net TiO_2_, being indeed similar to that of rGO. Thus, the photoluminescence intensity was diminished in the composites with respect to bare NPs, which is a usual behavior originated by the e^−^ acceptor and transport features given by the rGO support, resulting in suppressed charge recombination and less intense light emission. However, the composites with the lowest percentage of rGO showed the weakest photoluminescence intensity values, indicating that there is an optimal rGO content regarding the recombination rate. For the composite with the largest number of NPs, quantitatively more photoelectrons can be generated, thus resulting in a high number of potential recombinations and increased photoluminescence intensity. The deposited Pt on the TiO_2_ surface has been described to act as an electron sink, by trapping the electrons and further transferring them to the rGO support [25]. Comparing the applied Pt ratios, e.g., 0.1, 0.5, and 0.9 wt%, the lower the Pt loading, the weaker the photoluminescence response (Figure 5d). This result suggests that Pt can also act as a recombination center [49].

### 2.5. Photocatalytic Hydrogen Production

**Figure 6:**
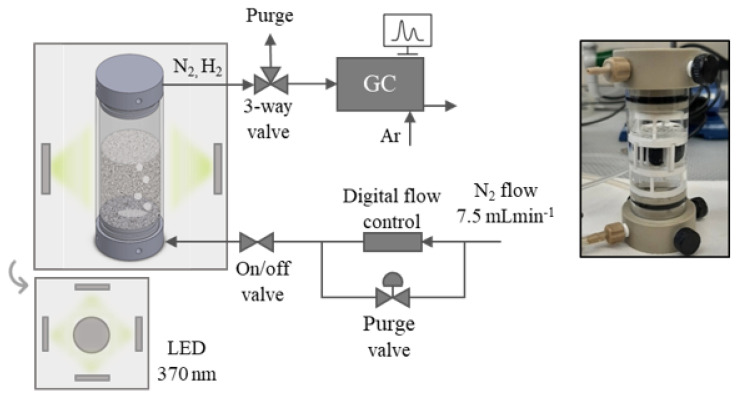
Scheme of the catalyst reactor setup and picture of the vessel involving the hand-made support holding the one-piece aerogel monolith.

Studies of photocatalytic H_2_ production were performed to evaluate the new catalytic system. For that, the applied conditions were first extensively examined and optimized. The most favorable conditions for the use of the new catalyst in a particular catalytic process would depend on both the character of the material (composition and structure) and the setup used. In this study, the analysis is focused on the optimization of the synthesized xPt/TiO_2_@rGO composite aerogel in regard to its photocatalytic activity in irradiated aqueous methanol solutions. The applied setup for the photocatalytic reaction is schematized in Figure 6. The studied parameters were catalyst reduction degree (from any to mild reduction), catalyst architecture (one-piece monolith or smashed aerogel), methanol concentration in the aqueous solution (from 0.01 to 1 v%), the added amount of catalyst to the reactor (from 0.03 to 2 g_NPs_L^−1^), Pt percentage in the catalyst (from 0 to 1 wt% in the Pt/TiO_2_ NPs), and NP ratio with respect to rGO (3:1 and 9:1).

In a typical experiment performed with the sample 0.9Pt/TiO_2_@rGO (3:1), the aerogel was recovered after the photocatalytic reaction and analyzed in regard to composition. The sample maintained the ratio 3:1 for NPs:rGO, thus indicating the lack of NP leaching, which opens the door for the recyclability of the material.

#### 2.5.1. Aerogel Reduction Degree

The straight use of GO monolithic aerogels with highly hydrophilic character in polar solvents causes the destruction of the macroscopic structure, provoked by strong electrostatic interactions with the solvent once it is immersed into the liquid. To avoid this drawback, the reduction in the GO phase to rGO was the applied solution in this work. The reduction step must be precisely controlled, since excessive reduction leads to highly hydrophobic aerogels that can suffer from low wettability when soaked in polar liquids, such as the water/methanol reaction medium used in this work. Hence, reaching an appropriate reduction degree of the GO composite aerogel is crucial to design an efficient catalyst in which the aqueous solution must easily travel inside the 3D structure [7]. In this work, a soft thermal treatment was applied for the reduction of the intermediate aerogel NPs@GO to NPs@rGO, e.g., 300 °C under a N_2_ atmosphere. A reduced aerogel with amphiphilic properties, involving graphitic hydrophobic regions and remaining hydrophilic oxygenated groups (hydroxyl and carboxyl), was thus synthesized. The decrease in hydrophilicity after reduction was depicted by water contact angle measurements, showing an increase in the contact angle for the reduced composite (58.9°) in comparison to the non-reduced (21.8°) (Figure S5). The relatively still high wettability found for the reduced composite is the consequence of the residual oxygenated groups and the involvement of the more hydrophilic TiO_2_ NPs, as well. 

**Figure 7:**
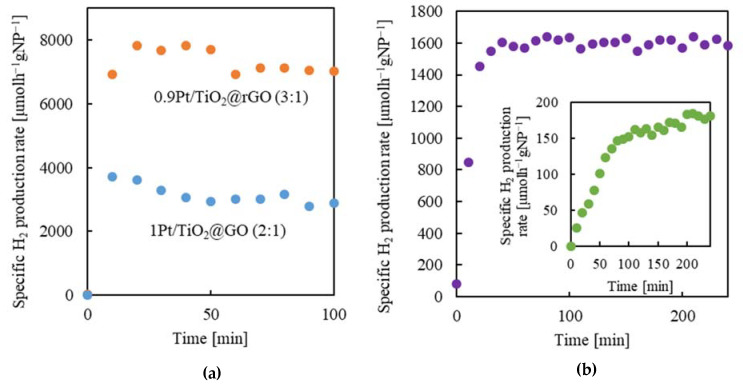
H_2_ production rate at different experimental conditions: (**a**) using the non-reduced 1Pt/TiO_2_@GO (2:1) and reduced 0.9Pt/TiO_2_@rGO (3:1) aerogels (reaction conditions: 0.5 *v*/*v* methanol/water, 0.5 g_NPs_L^−1^, smashed aerogel), and (**b**) using the 0.9Pt/TiO_2_@rGO aerogel as a one-piece monolith (green) and smashed (purple) (reaction conditions: 0.5 *v*/*v* methanol/water, 2g_NPs_L^−1^).

For the analyzed catalytic process, the necessity of reducing the GO support to rGO was established in a preliminary experiment in which the H_2_ evolution with time was compared for similar samples either non-reduced (1Pt/TiO_2_@GO (2:1)) or reduced (0.9Pt/TiO_2_@rGO (3:1)) under similar conditions in the catalytic reactor (0.5 *v*/*v* methanol/water solution, 0.5 g_NPs_L^−1^, and smashed aerogel). The obtained results indicate that the reduction step was necessary to improve the efficiency of the catalyst (Figure 7a).

Indeed, the H_2_ production in steady-state conditions was more than doubled for the reduced aerogel, increasing from 3280 µmol_H2_h^−1^g_NPs_^−1^ in the 1Pt/TiO_2_@GO (2:1) intermediate to 7070 µmol_H2_h^−1^g_NPs_^−1^ in the 0.9Pt/TiO_2_@rGO (3:1) aerogel. The reasons for the positive effect on the H_2_ production rate of the GO reduction are two-fold. On one hand, upon the removal of the oxygenated groups on the GO surface, the graphitic structure is partially restored, although in small spots due to defect generation [42,43]. Nevertheless, rGO would own more electrical pathways than GO, enhancing the conductivity of the matrix that plays a key role in transferring the photogenerated e^−^, thus preventing recombination and improving H_2_ production efficiency [50]. On the other hand, the more hydrophobic reduced structure favors the adsorption of the methanol sacrificial agent over water and maintains it close to the Pt/TiO_2_ NPs to boost h^+^ consumption, which was beneficial for production [26]. 

#### 2.5.2. Aerogel Architecture

Fabricating GO-based composite aerogels using scCO_2_ makes possible the creation of 3D monolithic meso/macroporous architectures. Preliminary tests were performed using a set-up designed for the straight use of the monoliths (Figure 6). Four of these cylinders were simultaneously used with a total weight of ca. 40 mg, which represents a catalytic NPs concentration of 2 g_NPs_L^−1^ in the reactor filled with 14 mL of a 0.5 *v*/*v* methanol/water solution. Under these conditions, a specific H_2_ production of 180 μmolh^−1^g_NP_^−1^ was reached at the steady state for the sample 0.9Pt/TiO_2_@rGO (3:1) (Figure 7b). Compared to the literature, this value is similar to those given in some of the published works (e.g., 100–400 μmolh^−1^g_NP_^−1^ using TiO_2_/Pt/rGO composites [25]), although it is considered to be in the low range of H_2_ production [26,27,29,30]. Definitely, the most important drawback of using monolithic one-piece aerogels in the catalytic experiment is relays in the small amount of sample exposed to light, so that during irradiation most of the Pt/TiO_2_ NPs in the interior part of the monolith remains inactive. Moreover, a long reaction time was needed to reach the steady state, in the order of 200 min, which was related to the slow diffusion of reactants in the monolithic aerogels with low permeability due to certain densification originated by shrinkage and high tortuosity. Diffusion is further hindered in non-stirred setups such as the one used in this work for the one-piece monoliths. Agitation was not used to avoid turbulence damage to the integrity of the monoliths during the measurements. Hence, in spite of the great prospect of applying monolithic aerogels to diminish the loss of active sites during recycling [7], the utilization of one-piece aerogel photocatalysts in liquid media appears to still face significant challenges [51]. 

To improve the catalytic activity of the aerogels, a second set of experiments was performed by first dispersing the reduced monoliths in small pieces in the aqueous methanol solvent, using for that soft and short sonication. The aim of the ultrasonic treatment was not to re-exfoliate the rGO flakes but to break the monoliths into pieces. The DLS characterization of the obtained dispersion gave a bimodal pattern with peaks at ca. 10 and 20 µm hydrodynamic sizes, representative of the size of the broken pieces of aerogel (Figure S6). After this treatment, the H_2_ production rate for the sample 0.9Pt/TiO_2_@rGO (3:1) increased to 1600 μmolh^−1^g_NPs_^−1^, with the particularity that this high rate was achieved after only 40 min (Figure 7b). The shortening in the required time to reach equilibrium is related to an increase in the catalyst permeability occurring for the small pieces with shorter throughout distances than the one-piece monoliths. Hence, permeability, even having similar absolute values for the smashed and one-piece aerogels is not the key parameter limiting the catalytic activity of the micrometric samples. The setup involving small pieces has the advantage that measurements can be performed under stirring, thus minimizing drawbacks related to reagent diffusion, adsorption, and desorption. Moreover, the small pieces of aerogel are continuously moving in the turbulences created by agitation, thus giving more chance for the catalytic NPs for being irradiated by light. All these factors lead to an enhancement in the H_2_ production rate.

#### 2.5.3. Methanol Concentration

**Figure 8:**
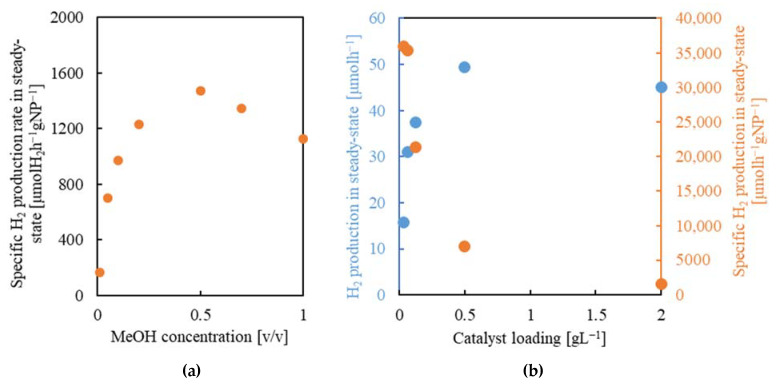
H_2_ production rate in steady-state conditions (60 min) for 0.9Pt/TiO_2_@rGO (3:1) smashed aerogel: (**a**) influence of methanol concentration (2 g_NPs_L^−1^), and (**b**) influence of NP concentration (0.5 *v*/*v* methanol/water), in which results are expressed as the non-specific (µmol_H2_ h^−1^, blue) and specific (µmol_H2_ h^−1^g_NPs_^−1^, orange) production rates.

Concerning methanol sacrificial agent concentration in the aqueous solution, diverse, even contradictory results have been published on TiO_2_-based systems used for photocatalytic H_2_ production. Actually, some of them conclude that methanol contributes less than its stoichiometric ratio to the overall H_2_ formation [52], while others confirm that the overall reaction can be described as the photoconversion of exclusively methanol [53]. In fact, it is expected that an increase in the methanol concentration in the aqueous solution results in enhanced H_2_ production, whether it comes either from the water/sacrificial agent or the sacrificial agent exclusively, due to the more effective scavenging of the photogenerated h^+^ by the alcohol. Water is known to play an important role in the complete oxidation of alcohol to CO_2_, making its presence necessary [54]. For instance, water has the ability to fasten the essential desorption of the reaction products from the catalyst surface, thus enhancing the reaction rate. As a consequence, after a certain increase in methanol concentration, the decrease in H_2_ production generally occurs due to the hindered adsorption of water on the catalyst surface already occupied by alcohol molecules. To optimize this parameter for the developed catalyst, methanol was applied in increased concentrations in the aqueous solution, from 10 to 100 v% (0.01 to 1 *v*/*v*). Experiments were performed with the smashed aerogel of sample 0.9Pt/TiO_2_@rGO (3:1) and a catalyst concentration of 2 g_NPs_L^−1^. The measured flow rates of the produced H_2_ at the steady state, e.g., at 60 min for each methanol concentration, are shown in Figure 8a. Initially, the increase in the methanol-to-water ratio favored the H_2_ production rate up to a maximum reached at a concentration of ca. 0.5 *v*/*v*. Thereafter, a further increase in the alcohol concentration results in a smooth decrease in the H_2_ evolution. Commonly, a behavior of a sharp decline in H_2_ generation has been observed for similar catalysts [55,56]. The lack of severe decrease, caused by excess methanol on the catalytic efficiency of the studied composite, is here related to the swelling characteristics of the used rGO support. Although the photocatalytic reaction is induced by the photogenerated h^+^ and e^−^ on the Pt/TiO_2_ NPs, the swelling of the rGO support is influenced by the polarity of the solvent, which affects the interaction between the dispersed active sites on the rGO surface and the reactants. Applying methanol in high concentrations would enhance the swelling of the aerogel pieces since methanol interaction with the hydrophobic graphitic regions in the rGO support would be stronger than for water [57]. The swelled structure would allow better accessibility for the reactants to the attached NPs. Hence, the above-mentioned adverse effects of the excess of methanol are somewhat compensated by aerogel swelling, and the H_2_ production is maintained at a relatively high level in all the studied ranges of alcohol concentration. 

#### 2.5.4. Catalyst Concentration

The effect of the catalyst loading, referring to the number of NPs, was investigated in the concentration interval of 0.03 to 2 g_NPs_L^−1^ (equivalent to 0.04–2.67 g_aerogel_L^−1^) for the 0.9Pt/TiO_2_@rGO (3:1) smashed aerogel dispersed in 0.5 *v*/*v* water/methanol solution. The production rate of H_2_ was measured at a steady state (60 min) (Figure 8b). In the studied interval of concentration, the H_2_ flow rate, expressed as the specific value, e.g., normalized to the catalyst NPs weight (µmol_H2_h^−1^g_NPs_^−1^), was very high at low catalyst loading (0.03–0.125 g_NPs_L^−1^), and then substantially decreased at high concentrations (>0.5 g_NPs_L^−1^). However, this result, which could be taken at the first instance as an indication of the benefits of working at a very low concentration of catalyst, is just a mathematical artifact since, in fact, the total amount of the produced H_2_ can be considered as being in the low range. The representation of the catalytic data as a function of the non-specific H_2_ production rate (µmol_H2_h^−1^) indicates that the total amount of evolved H_2_ sharply increases with the catalyst loading up to a value of ca. 0.5 g_NPs_L^−1^. Thereafter, H_2_ production slightly decreases by increasing catalyst concentration. This decrease is likely due to light blocking by an excess of dark solid catalyst dispersion [58]. 

#### 2.5.5. Catalyst Composition

##### Platinum Content

**Figure 9:**
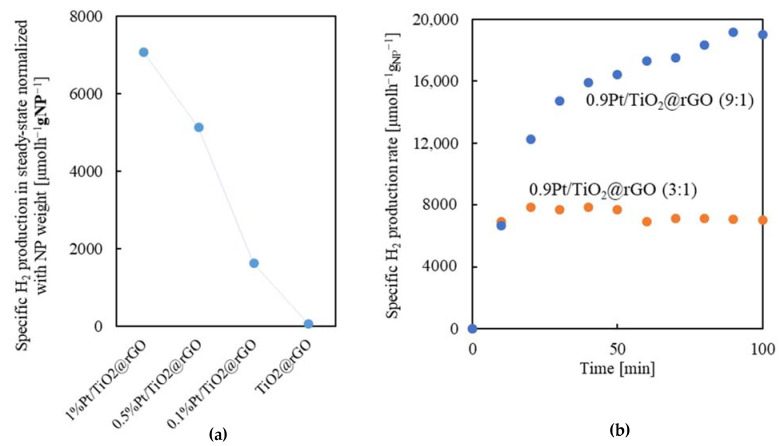
Specific H_2_ production rate for smashed aerogel samples: (**a**) xPt/TiO_2_@rGO (3:1) with different Pt content, and (**b**) 0.9Pt/TiO_2_@rGO with 3:1 and 9:1 NP:rGO ratios. Reaction conditions: 0.5 *v*/*v* methanol/water, 0.5 g_NPs_L^−1^.

Regarding the catalyst composition, one important parameter for regulating the photocatalytic activity in the reaction of H_2_ production is the amount of Pt added to the TiO_2_ NPs. The presence of Pt is necessary for suppressing the recombination of the photogenerated e^−^ and h^+^ in the TiO_2_ semiconductor, thus enhancing the formation of H_2_. Intimate Pt–TiO_2_ contact at the interphase is also necessary to maximize the H_2_ production efficiency [59]. To analyze this parameter, a series of experiments was performed with smashed xPt/TiO_2_@rGO (3:1) aerogels with four different values of Pt content in the NPs: 0, 0.1, 0.5, and 1.0 wt%. In previous works involving Pt/TiO_2_/rGO systems, the proportion of the noble metal is also within this range, typically 0.4–1% [17,25,26,29], which facilitates data comparison. Measurements were carried out at the optimal reaction conditions previously established, e.g., 0.5 *v*/*v* water/methanol and smashed catalyst with a concentration of 0.5 g_NPs_L^−1^. Primary tests indicated that without the addition of the noble metal (sample TiO_2_@rGO), the H_2_ evolution was negligible, with a value of only 60 µmol_H2_h^−1^g_NPs_^−1^. This result corroborates previous findings pointing to the inactivity of TiO_2_ NPs without the use of a co-catalyst [60]. In the studied range of noble metal loading, the H_2_ production increases concomitantly with Pt content (Figure 9a). The decline in H_2_ evolution was smoother when the Pt content was decreased from 0.9 to 0.5 wt% than from 0.5 to 0.1 wt%. This observation indicates that, although the overall H_2_ production was the highest with the sample of 0.9 wt% Pt content in TiO_2_, the total amount of noble metal can be halved without losing significant activity. This is an important result since Pt is the most expensive component of the catalyst; therefore, the amount of noble metal incorporated into the composite would be crucial in any industrial process and must be reduced as much as possible. The design of a catalyst involving an important reduction in the use of Pt is currently an important goal targeted by the European Commission [61]. 

##### NP:rGO Ratio

The influence of modifying the NPs:rGO ratio in the 0.9Pt/TiO_2_@rGO composite was investigated by increasing this value from 3:1 to 9:1. This modification resulted in a 2.7-fold enhancement in the H_2_ production efficiency, from 7070 to 18,800 µmol_H2_h^−1^g_NPs_^−1^ at the steady state (Figure 9b). The foremost effect of increasing the NP loading in the composite was to enhance the light-harvesting of the catalyst since, statistically, the probability for the UV radiation to contact active centers rises. On the contrary, the large amount of rGO in the aerogel with the lowest number of NPs shields an important portion of the active centers, hindering overall catalyst activity [62]. Moreover, the steady state in H_2_ evolution was reached at 10 and 90 min for the 3:1 and 9:1 samples, respectively. Increasing the rGO percentage in the composite should raise the hydrophobic character of the aerogel, thus improving the accessibility of the methanol vs. water in the pores. Excess amounts of methanol close to the photogenerated holes facilitate the fast h^+^ trapping by the alcohol, such that in the initial phase of the reaction, the H_2_ production already reaches its limit [57]. Hence, the sample with the lowest proportion of NPs has the advantage of rapidly reaching the steady state, while the sample with the higher proportion revealed the largest H_2_ production rate. One point that should be underlined particularly is that the value of the H_2_ production rate obtained for the 0.9Pt/TiO_2_@rGO (9:1) catalyst exceeds ca. 2–10 times the values published for similar systems using aqueous methanol solutions as reaction media and involving Pt/TiO_2_/rGO in the catalyst [25,29]. 

## 3. Conclusions

Three-dimensional porous Pt/TiO_2_@GO and Pt/TiO_2_@rGO composite aerogels were prepared using the one-step low-temperature green supercritical CO_2_ method. The produced aerogels are intended for the photocatalytic production of H_2_ from aqueous methanol solutions. For this application, optimal working operational conditions resulting in the highest H_2_ production rate were settled as a 0.5 g_NPs_L^−1^ catalyst concentration in a 0.5 *v*/*v* methanol/water reaction solution. A two-fold increase in the H_2_ production was observed when the GO support was mildly reduced to rGO, an effect assigned to the generation of new electronic pathways upon the partial restoration of the graphene network, and the favored adsorption of the methanol in the reduced structure. The moderate H_2_ production rate observed when a one-piece monolith was used (180 μmolh^−1^g_NP_^−1^) was significantly improved (ca. 10-fold) when the aerogels were broken into small pieces (1600 μmolh^−1^g_NP_^−1^), shortening also the time needed to reach the equilibrium from 200 to only 40 min. This enhancement is the result of the improved light exposure of the active sites and increased reagent and product diffusion. Increasing the NP:rGO ratio from 3:1 to 9:1 caused a 2.7-fold increase in the H_2_ evolution due to the reduced amount of shielded, and thus inactive, NPs. Regarding the catalyst composition, low Pt percentages, in the order of 0.9–0.5 wt%, can be used, still giving a high H_2_ production rate. In the most favorable conditions, an H_2_ production of 18,800 µmolh^−1^g_NP_^−1^ was measured for the 0.9Pt/TiO_2_@rGO (9:1) aerogel catalyst in aqueous methanol, which is remarkably high compared to the reported similar Pt/TiO_2_/rGO systems.

## 4. Materials and Methods

### 4.1. Materials

For the preparation of the Pt/TiO_2_ NPs, chloroplatinic acid hexahydrate (H_2_PtCl_6_.6H_2_O) and TiO_2_ NPs (AEROXIDE P25, ca. 20 nm), provided by Alfa Aesar and Evonik, respectively, were used. For the aerogel preparation, a GO water dispersion of 4 mgmL^−1^, supplied by Graphenea Inc. (Spain), was employed. Ethanol and methanol were purchased from Carlo Erba. Liquid CO_2_ (99.95 wt%). N_2_ and H_2_ gasses were delivered by Carburos Metálicos S.A. 

### 4.2. Synthetic Methods

#### 4.2.1. Preparation of NPs of xPt/TiO_2_ Composite

NPs of Pt/TiO_2_ with different Pt contents were prepared following a reported incipient impregnation deposition and followed by reduction methodology [63]. Briefly, a weighted amount of H_2_PtCl_6_.6H_2_O, e.g., 2.7, 13, or 26 mg, was dissolved in 2.5 mL of ultrapure water and used to obtain Pt/TiO_2_ composites with 0.1, 0.5, and 1.0 wt% Pt contents, respectively. Each Pt solution was added dropwise to 1 g of TiO_2_ NPs at a rate of 8.33 μLmin^−1^, achieved by using a peristaltic pump, while the resulting slurry was continuously sonicated. After that, the deposited suspension was kept under sonication for 2 h. The dense dispersion was dried at 100 °C overnight in an air oven. The recovered powder was treated at 200 °C in a tubular oven, increasing the temperature with a heating ramp of 10 °C min^−1^, first under N_2_ for 1 h and then reduced under a H_2_ flow of 50 mL min^−1^ for 3 h. A grey powder was obtained and named as xPt/TiO_2_, where x indicates the added Pt weight content in percentage.

#### 4.2.2. Preparation of xPt/TiO_2_@rGO Composite Aerogels

For the composite aerogels, a suspension of GO in ethanol with an adjusted concentration of 3.5 mg mL^−1^ was first prepared from water dispersion following a reported protocol [36]. Weighted amounts of bare TiO_2_ and composed xPt/TiO_2_ NPs were dispersed by sonication in aliquots of 1 mL of the GO-ethanol suspension to obtain weight ratios of 2:1 and 6:1 for NPs:GO. The suspensions were added to assay tubes of ca. 1 cm diameter and 2 mL volume and placed in a 200 mL high-pressure reactor (TharProcess). The aerogels were prepared by drying the suspensions with scCO_2_ in the batch mode, keeping the autoclave at 200 bar and 45 °C for 48 h (Figure 1a) [36]. Finally, the CO_2_ was slowly released from the reactor under isothermal conditions. The xPt/TiO_2_@GO samples were recovered as one-piece cylindrical monoliths. The reduction of the recovered 3D GO aerogels to rGO was carried out in a tubular oven at 300 °C under N_2_ flow. To reach the target temperature, a heating ramp of 5 °C min^−1^ was used, upholding the temperature for 20 min after reaching 100 and 200 °C, and then maintaining it for 2 h at 300 °C. The reduced xPt/TiO_2_@rGO aerogels were also recovered as one-piece monoliths and used either as-synthesized or smashed into small pieces.

### 4.3. Characterization

The platinum content in the xPt/TiO_2_@rGO aerogels was quantified by inductively coupled plasma mass spectrometry (ICP-MS, Agilent 7700x) after digesting the samples in hydrochloric, nitric, and hydrofluoric acids (3:1:0.5 *v*/*v*). The structural characterization of the prepared NPs and the reduced composite aerogels was performed by powder X-ray diffraction (PXRD) in a Siemens D5000, using the Cu Kα incident radiation with a step scan of 0.02° in the 2θ 5–40° range. The size of the NPs was estimated using PXRD data and the Scherrer equation. Surface functional groups were studied by Fourier transform infrared (FTIR) spectroscopy (Jasco 4700 Spectrophotometer), after the dispersion of the samples in potassium bromide (KBr). Raman spectra were recorded to ascertain the reduction in GO by using an excitation wavelength of 532 nm. The morphology of the composite aerogels and the size of the NPs, as well as their degree of dispersion on the rGO platelets, were investigated by scanning (SEM, Quanta FEI 200) and transmission (TEM, JEOL 1210) electron microscopies. The BET (Brunauer, Emmet, Teller) surface area (S_a_), the BJH (Barrett, Joyner, and Halenda), and cumulative adsorption pore volume (V_p_) were determined by collecting N_2_ adsorption/desorption isotherms at 77 K (ASAP 2020, Micromeritics Inc., Norcross, GA, USA), after degassing the samples at 393 K for 20 h. For the smashed aerogels, dynamic light scattering (DLS Coulter LS230) was used to study the hydrodynamic size of the aerogel broken pieces dispersed in methanol/water (0.5 *v*/*v*). The wettability of the reduced and non-reduced composites was investigated by water contact angle measurement (Biolin Sci. Attension Theta Lite) after preparing a compressed pellet with the monoliths. The optical properties of the aerogels were investigated by UV-VIS diffuse reflectance (Jasco V-560) and photoluminescence (Jasco FP-8300) spectroscopies using the smashed solid samples. For the UV experiments, barium sulfate (BaSO_4_) powder was used as blank.

### 4.4. Photocatalytic H_2_ Production

The photocatalytic activity of the synthesized composite aerogels for H_2_ production was tested by immersing the reduced monoliths, either as recovered in one piece or ultrasonically smashed, in an aqueous methanol solution. For the one-piece samples, a specifically designed basket composed of poly-lactic acid (PLA) polymer was fabricated with 3D printing to hold four monoliths and prevent them from floating during the reaction. The basket was settled inside a cylindrical glass reactor of 20 mL that was filled with 14 mL of a methanol/water mixture (Figure 6). For the tests with the smashed aerogels, four monoliths were added to the methanol/water solution, gentile sonicated for 5 min in an ultrasonic bath, and poured into the 20 mL reactor vessel without the basket but under mechanical stirring (100 rpm). The methanol percentage in the methanol/water mixture was varied from 0.01 to 1 *v*/*v*. Different aerogel catalyst concentrations, from 0.04–2.67 g_aerogel_L^−1^ (equivalent to 0.03 to 2 g_NPs_L^−1^) were also tested. Before starting the catalytic reaction, the system was purged with N_2_, and a stream of this inert gas was continued at a rate of 7.5 mL min^−1^ during the entire experiment. The reaction mixture was irradiated with a four visible-LED system placed at a 4 cm distance from the cylindrical reactor wall. The emission spectrum is shown in the SI (Figure S7). The average nominal irradiance of each LED was 45.0 mW cm^−2^, determined by using a UV–vis spectroradiometer (OceanOptics USB2000+). The H_2_ production rate was analyzed online every 10 min in the headspace of the reactor by using a gas chromatograph (Inficon 3000 MicroGC). The results are expressed referring to either the rate of H_2_ production [µmol_H2_h^−1^] or the rate per mass of the NPs [µmol_H2_h^−1^g_NPs_^−1^]. Steady-state H_2_ production data recorded after 60 min were calculated for all the analyses performed with the smashed monoliths.

## Data Availability

Not applicable.

## Supplementary Materials

The following supporting information can be downloaded at: https://www.mdpi.com/article/10.3390/gels8110719/s1, Figure S1: XRD spectra of the non-reduced 1Pt/TiO_2_@GO and reduced 0.9Pt/TiO_2_@rGO (3:1) composites, Figure S2: TEM images of: (a) bare TiO_2_, and (b) 0.9Pt/TiO_2_ NPs obtained by subjecting 1Pt/TiO_2_ NPs to the reduction treatment; Figure S3: N_2_ adsorption/desorption isotherms for the 0.9Pt/TiO_2_@rGO sample compared to the precursors GO and 1Pt/TiO_2_ NPs.; Figure S4: BJH volumetric pore size distribution calculated from the adsorption branch of the isotherm; Figure S5: Contact angle measurement of: (a) 0.9Pt/TiO_2_@rGO and 1Pt/TiO_2_@GO composites, measured with a water droplet; Figure S6: DLS analysis of the 1Pt/TiO_2_-rGO (3:1) aerogel dispersed in an aqueous methanol solution (0.5 *v*/*v*); Determination of the band gap energy of the photocatalysts; Figure S7: Emission spectrum of the used light source.
