# Supplementary material for: Photocatalytic Hydrogen Production Using Porous 3D Graphene-Based Aerogels Supporting Pt/TiO2 Nanoparticles
Source: Gels. 2022 Nov 7;8(11):719. doi: 10.3390/gels8110719 (PMC9689606; doi:10.3390/gels8110719)
Supplement: Supplementary file 1 [file gels-08-00719-s001.zip › gels-1973710-supplementary.pdf]

Supplementary Information

Photocatalytic hydrogen production using porous 3D graphene-based aerogels supporting Pt/TiO_2 nanoparticles

Márta Kubovics[1],* Cláudia G. Silva[2,3], Ana M. López-Periago[1], Joaquim L. Faria[2,3], and Concepción Domingo[1]*

[1]Instituto de Ciencia de Materiales de Barcelona. CSIC, Campus UAB s/n, 8193 Bellaterra, Spain

[2]LSRE-LCM - Laboratory of Separation and Reaction Engineering – Laboratory of Catalysis and Materials, Faculty of Engineering, University of Porto, Rua Dr. Roberto Frias, 4200-465 Porto, Portugal

[3]ALiCE - Associate Laboratory in Chemical Engineering, Faculty of Engineering, University of Porto, Rua Dr. Roberto Frias, 4200-465 Porto, Portugal

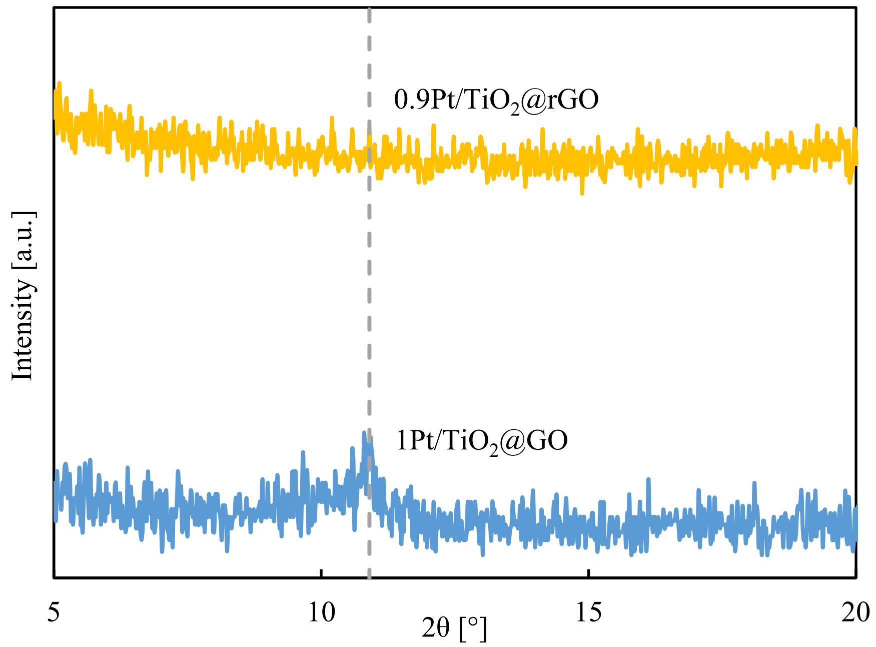

Figure S1. XRD spectra of the non-reduced 1Pt/TiO_2@GO and reduced 0.9Pt/TiO_2@rGO (3:1) composites. The small peak representative of GO is marked with a grey dashed line.

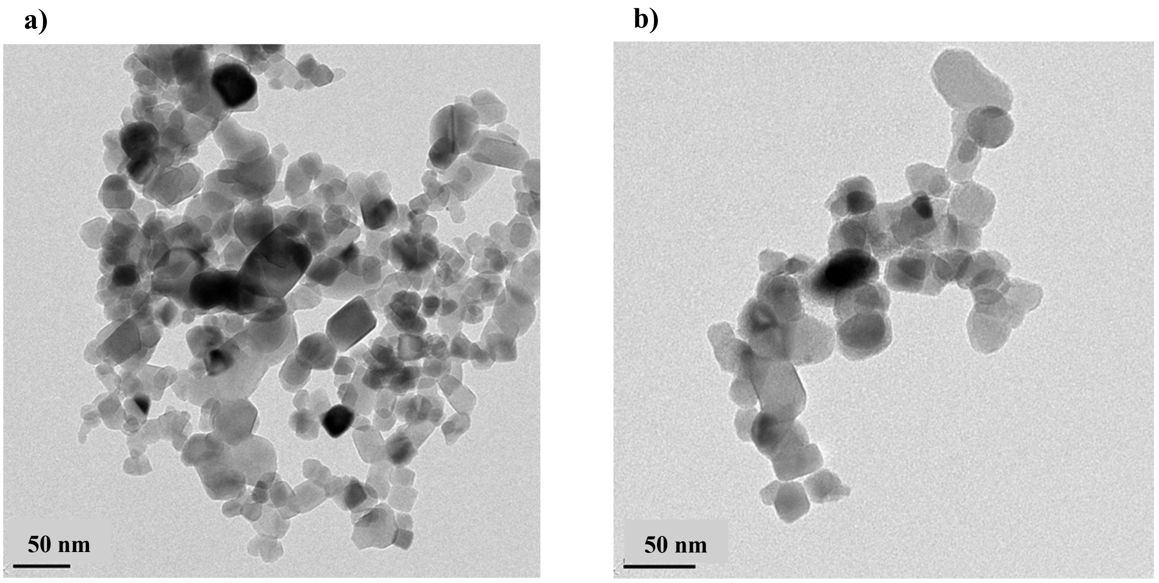

Figure S2. TEM images of: (a) bare TiO_2, and (b) 0.9Pt/TiO_2 NPs obtained by subjecting 1Pt/TiO_2 NPs to the reduction treatment.

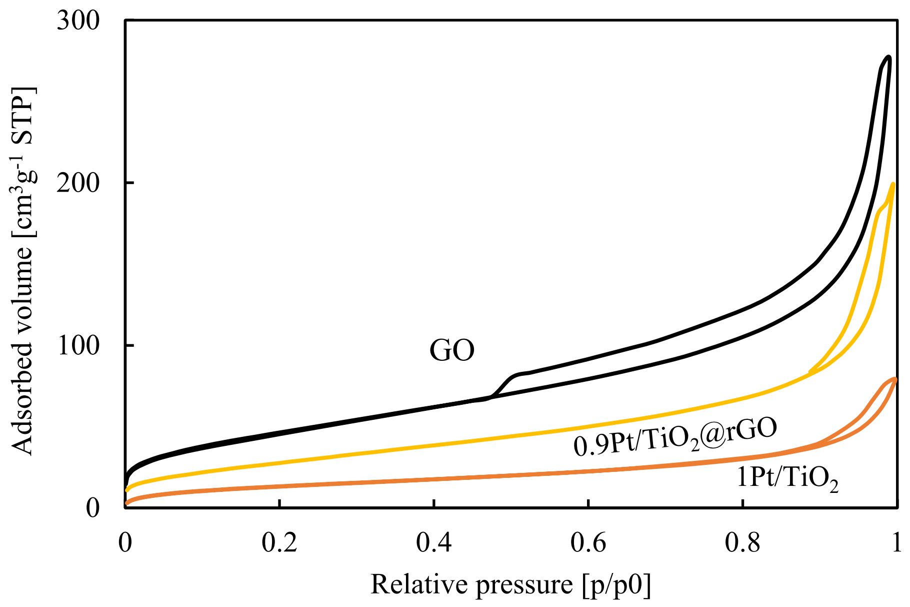

Figure S3. N_2 adsorption/desorption isotherms for the 0.9Pt/TiO_2@rGO sample compared to the precursors GO and 1Pt/TiO_2 NPs.

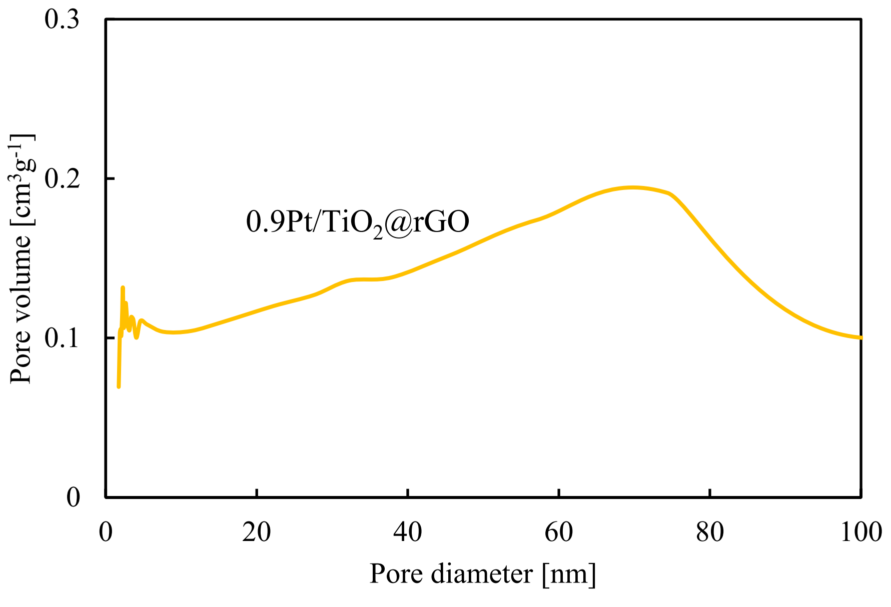

Figure S4. BJH volumetric pore size distribution calculated from the adsorption branch of the isotherm.

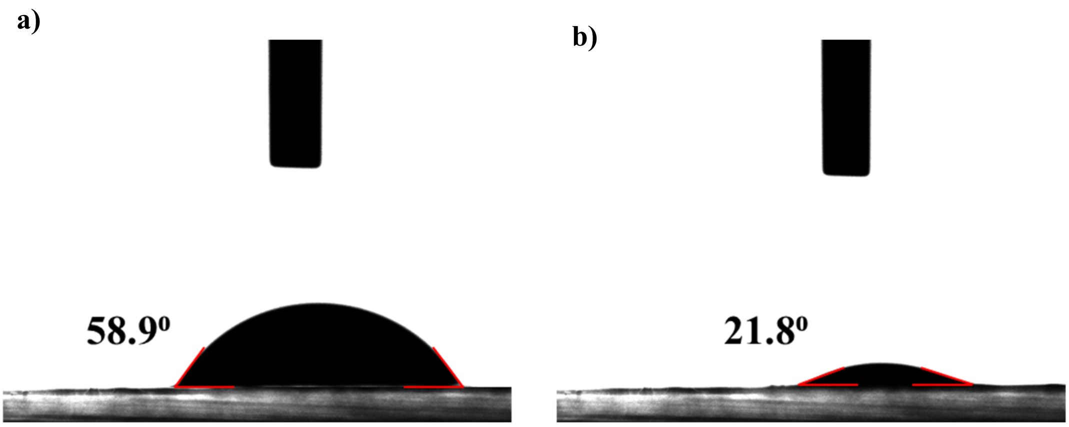

Figure S5. Contact angle measurement of: (a) 0.9Pt/TiO_2@rGO and 1Pt/TiO_2@GO composites, measured with a water droplet.

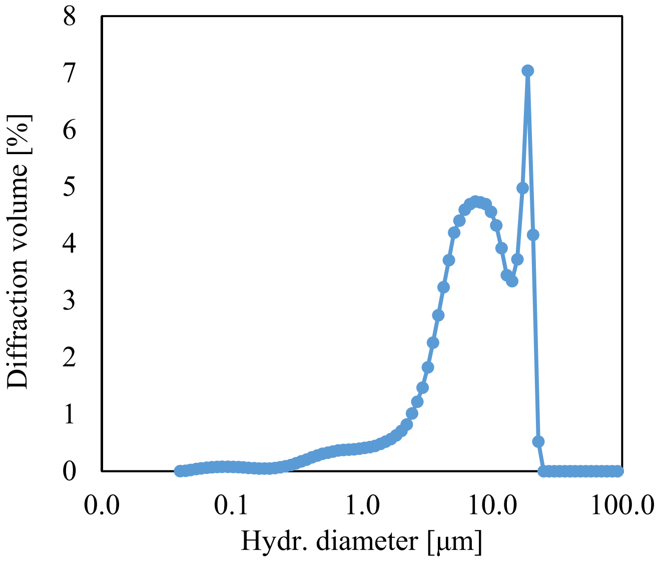

Figure S6. DLS analysis of the 0.9Pt/TiO_2@rGO (3:1) aerogel dispersed in an aqueous methanol solution (0.5 v/v). Data measured after 5 min of ultrasound treatment.

Determination of the band gap energy of the photocatalysts[1]

The energy-dependent adsorption coefficient of the semiconductor is determined by the following equation:

$$(\alpha \cdot h\vartheta)^{\frac{1}{\gamma}} = B(h\vartheta - E_g)$$

Where α is the absorption coefficient, h is the Planck constant, ϑ is the frequency of the photon, B is a constant, and E_g is the band gap energy. In the case of the TiO_2 semiconductor, γ = ½, as the electron transition is indirect.

The measure reflectance spectra are transformed to F(R∞) by applying the Kubelka–Munk function.[2] By approximating the absorption coefficient with F(R∞), the following form is obtained:

$$(F(R_\infty) \cdot h\vartheta)^{\frac{1}{2}} = B(h\vartheta - E_g)$$

Plotting $(F(R_\infty) \cdot h\vartheta)^{\frac{1}{2}}$ *vs.* the photon energy gives the Tauc plot (shown in the main text), with a region displaying a steep linear increase in absorption, characteristic of the semiconductor. From the x-axis intercept of the extrapolated line fitted to the linear region of the plot, the estimated band gap energy can be determined.

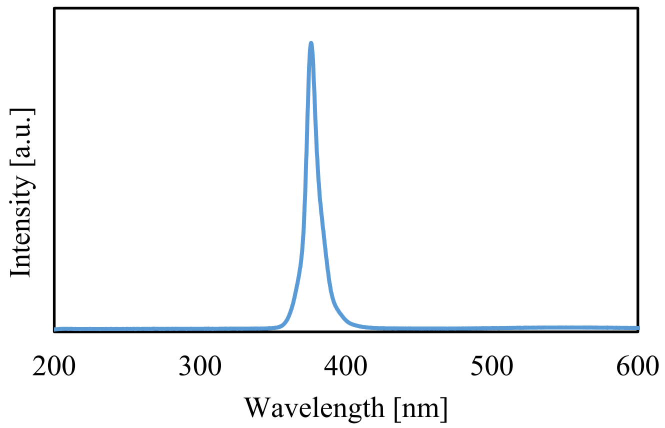

Figure S7. Emission spectrum of the used light source.

References

1. Makuła, P.; Pacia, M.; Macyk, W. How To Correctly Determine the Band Gap Energy of Modified Semiconductor Photocatalysts Based on UV-Vis Spectra. *Journal of Physical Chemistry Letters*, **2018**, *9*, 6814–6817, doi: 10.1021/acs.jpclett.8b02892.

2. Munk, P.; Kubelka, F. A Contribution to the Optics of Pigments, *Z. Tech. Phys.*, **1931**, *12*, 593–601.
